# Allosteric Modulation of Muscarinic Acetylcholine Receptors

**DOI:** 10.3390/ph3092838

**Published:** 2010-08-30

**Authors:** Jan Jakubík, Esam E. El-Fakahany

**Affiliations:** 1 Institute of Physiology, Academy of Sciences of the Czech Republic, Vídeňská 1083, 142 00 Praha, Czech Republic; 2 Division of Neuroscience Research in Psychiatry, University of Minnesota Medical School, Minneapolis, MN 55455, USA

**Keywords:** muscarinic acetylcholine receptors, allosteric modulation, Alzheimer’s disease, schizophrenia

## Abstract

An allosteric modulator is a ligand that binds to an allosteric site on the receptor and changes receptor conformation to produce increase (positive cooperativity) or decrease (negative cooperativity) in the binding or action of an orthosteric agonist (e.g., acetylcholine). Since the identification of gallamine as the first allosteric modulator of muscarinic receptors in 1976, this unique mode of receptor modulation has been intensively studied by many groups. This review summarizes over 30 years of research on the molecular mechanisms of allosteric interactions of drugs with the receptor and for new allosteric modulators of muscarinic receptors with potential therapeutic use. Identification of positive modulators of acetylcholine binding and function that enhance neurotransmission and the discovery of highly selective allosteric modulators are mile-stones on the way to novel therapeutic agents for the treatment of schizophrenia, Alzheimer’s disease and other disorders involving impaired cognitive function.

## 1. Introduction

Acetylcholine was postulated as a possible neurotransmitter by Henry Hallett Dale in 1914 [1] and confirmed as such by Otto Loewi in 1921 [2]. Acetylcholine is a neurotransmitter in both the peripheral nervous system and central nervous system (CNS) in humans and many organisms, including invertebrates. Acetylcholine acts through two classes of receptors – muscarinic and nicotinic acetylcholine receptors. While, muscarinic acetylcholine receptors are metabotropic receptors, nicotinic receptors are ligand-gated cationic channels. Muscarinic receptors are members of the G-protein-coupled receptor (GPCR) family A. To date, five distinct subtypes of muscarinic acetylcholine receptors (M_1_–M_5_) have been cloned and sequenced [3]. M_1_, M_3_ and M_5_ subtypes preferentially activate phospholipase C and calcium mobilization through G_q/11_ whereas M_2_ and M_4_ receptors inhibit the activity of adenyl cyclase or modulate conductance of ion channels through the G_i/o_ family of G-proteins [4]. Cholinergic signaling plays a critical role in a wide variety of CNS and peripheral functions including learning, memory and attention mechanisms, motor control, nociception, regulation of sleep-wake cycles, cardiovascular function, renal and gastrointestinal functions, and many others. A wide variety of CNS disorders including Alzheimer's disease, Parkinson's disease, schizophrenia, epilepsy, sleep disorders, neuropathic pain, and others involve malfunction of cholinergic transmission. Evidence suggests that cholinergic neurotransmission in the forebrain regions and cholinergic involvement in learning and memory are mediated primarily by muscarinic receptors [5]. This implies that agents that selectively modulate the activity of specific subtypes of these receptors may have therapeutic potential in abovementioned pathological states [6,7].

## 2. Role of Muscarinic Receptors in Psychiatric and Neurological Disorders

The most important role of muscarinic receptor-mediated cholinergic neurotransmission in the CNS relates to cognitive function. Disruptions of the cholinergic system in rodents revealed an important function in short and long-term memory processing [8,9]. Clinical studies with muscarinic receptor agonists demonstrated the potential of this class of compounds to reverse cognitive deficits associated with disrupted cholinergic neurotransmission. For instance, in Alzheimer’s clinical studies, the inhibitor of acetylcholinesterase physostigmine and the muscarinic receptor agonist arecoline have been shown to improve cognition [10]. Several lines of evidence indicate that the most prominent adverse effects of acetylcholinesterase inhibitors and non-selective muscarinic agonists are mediated by activation of peripheral M_2_ and M_3_ receptors. They include bradycardia, gastrointestinal distress, excessive salivation, and sweating [11,12]. In contrast, selective distribution of M_1_ receptors in the forebrain and the deleterious effects of M_1_ antagonists on memory and learning indicate a primary role of this subtype of muscarinic receptors in cognition, attention mechanisms, and sensory processing [7]. M_1_ muscarinic receptor agonists promote non-amyloidogenic processing of the amyloid precursor protein (APP) (cleavage by α-secretase in the middle of the Aβ sequence) that prevents formation of noxious amounts of Aβ fragments [13,14]. This shift in APP processing is mediated by ERK1/2 and PKC activation [13]. Involvement of muscarinic receptors in decelerating progression of the disease is supported by demonstration of accelerated amyloid pathology in Parkinson´s disease patients treated with muscarinic receptor antagonists [15]. Similarly, the decline in strength of muscarinic signal transduction in the cerebral cortex that develops along with accumulation of soluble β-amyloid and markedly precedes behavioral impairments and amyloid pathology has been demonstrated in a transgenic mouse model of Alzheimer´s disease [16,17].

Another psychiatric condition that involves cholinergic transmission is schizophrenia. Schizophrenia is a diagnosis that covers a set of disorders of different etiologies with the same symptoms. This disorder can be divided based on characteristics of negative symptoms to deficit and non-deficit ones or according to DSM-IV (The Diagnostic and Statistical Manual of Mental Disorders) to paranoid, disorganized, catatonic, undifferentiated, and residual types. One of the hypotheses for the etiology of schizophrenia based on involvement of muscarinic receptors surfaced from clinical observations that anticholinergic agents, such as scopolamine, were shown to induce a psychotic state similar to schizophrenia and exacerbate symptoms in schizophrenic patients. Moreover, clinical trials provided evidence that muscarinic agonists were moderately effective as neuroleptic agents [18,19]. In neuropathological studies it has been shown that levels of both M_1_ and M_4_ receptors are reduced in the prefrontal cortex, hippocampus, caudate and putamen in postmortem samples from schizophrenic patients [20,21,22,23,24]. Knock-out studies have been employed to further link muscarinic receptors to the pathology of schizophrenia [11,25]. From studies in knockout mice, the M_1_ receptor subtype has been viewed as the most likely candidate for mediating effects on cognition, attention mechanisms, and sensory processing. The M_4_ receptor is localized in dopamine-rich brain regions (the mesolimbic dopaminergic pathway), and regulates dopamine levels in this region [26].

## 3. Therapeutic Potential of Muscarinic Allosteric Modulators

An allosteric modulator is a ligand that binds to an allosteric (secondary) site on the receptor and changes receptor conformation to produce increase (positive cooperativity) or decrease (negative cooperativity) in the binding or action of an orthosteric agonist (e.g., acetylcholine). As opposed to classical agonists, positive allosteric modulators of natural neurotransmitters have the following advantages: (1) they mimic neurotransmission under physiological conditions— they preserve time and space pattern of the signal; (2) greater subtype selectivity can be obtained as positive or negative modulation of a given receptor subtype combined with neutral cooperativity (no change in binding or action of classical agonist upon binding of the allosteric modulator) at other subtypes; (3) the magnitude of the effects of an allosteric modulator on action of a natural neurotransmitter is limited by the magnitude of allosteric interaction [27]. The effects of an allosteric modulator reach a maximum that is not exceeded by increasing the dose; (4) positive modulation at one subtype may be combined with negative modulation at another. For example, common cholinergic synapses in the forebrain contain M_1_ postsynaptic receptors and M_2_ presynaptic receptors that mediate feedback inhibition of acetylcholine release. Positive allosteric modulation of postsynaptic M_1_ receptors and negative modulation of presynaptic M_2_ receptors by a given allosteric modulator would have desired synergistic effects in enhancing cholinergic neurotransmission; (5) allosteric binding sites have not faced the same evolutionary pressure as orthosteric sites to accommodate an endogenous neurotransmitter and thus may show greater divergence among subtypes. This endows allosteric modulators with higher selectivity among receptor subtypes. 

## 4. First Allosteric Modulators of Muscarinic Receptors

**Figure 1:**
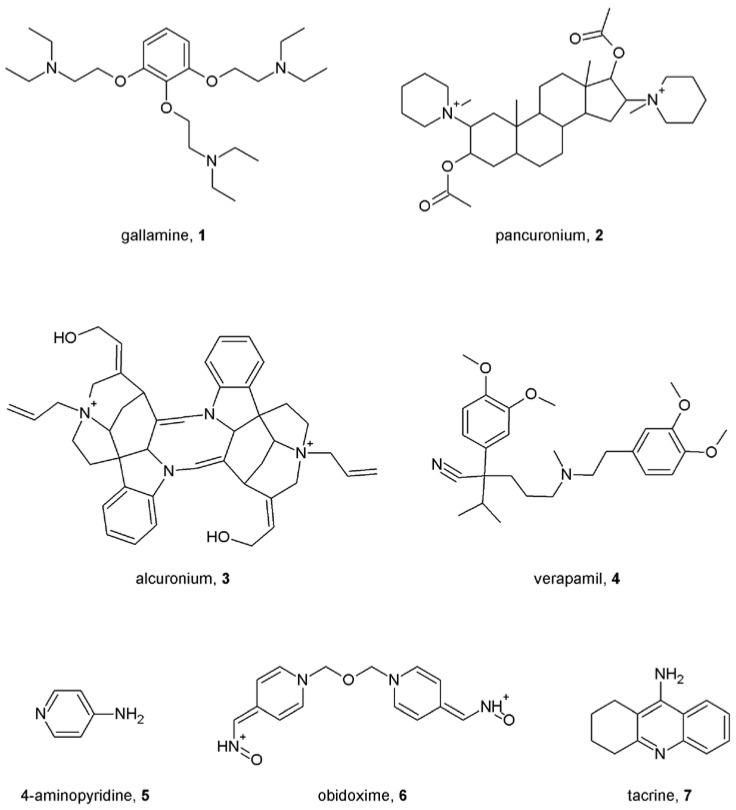
Structures of prototypical allosteric modulators of muscarinic receptors.

In the pioneering work by Clark and Mitchelson, gallamine (**1**; Figure 1) was found to inhibit the action of acetylcholine and carbachol on the heart atrium in functional experiments [28]. The concentration-response curves were shifted to the right by gallamine but the magnitude of the progressive shifts diminished with increasing concentrations of gallamine. When the action of acetylcholine on the heart was evaluated in the combined presence of gallamine and the antagonist atropine, the concentration ratio was less than that expected from experiments with gallamine or atropine alone. These observations led to the conclusion that the action of gallamine is allosteric. The allosteric nature of the gallamine action was then confirmed in binding studies [29] where it slowed down dissociation of the radiolabeled orthosteric antagonist *N*-methylscoplamine (NMS). Paradoxically, gallamine is a neuromuscular blocker. By definition, neuromuscular blockers are nicotinic acetylcholine receptor antagonists, but many of them ([e.g., gallamine (**1**), pancuronium (**2**) and alcuronium (**3**); Figure 1] have high affinities for the M_2_ receptor and act allosterically at that receptor [30,31]. Advances in radioligand binding techniques and the availability of muscarinic radioligands with high specific to non-specific binding ratio such as *N*-methylscopolamine (NMS) and quinuclidinyl benzilate (QNB) led to identification of many allosteric modulators. These included blockers of acetylcholinesterase, another acetylcholine binding protein, and channel blockers, for example, the L-calcium blocker verapamil (**4**) [32], the potassium channel inhibitor 4-aminopyridine (**5**) [33] and the acetyl-cholinesterase inhibitors obidoxime (**6**) and its bisquaternary pyridinium analogues [34] and tacrine (**7**) [35] (Figure 1).

## 5. Nature of Allosteric Modulators

**Figure 2:**
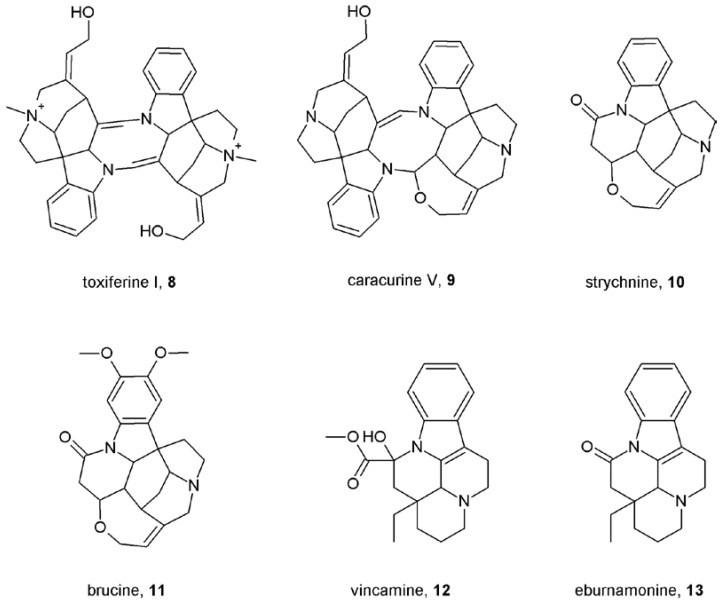
Structures of toxiferous, strychnos and vinca alkaloids.

**Figure 3:**
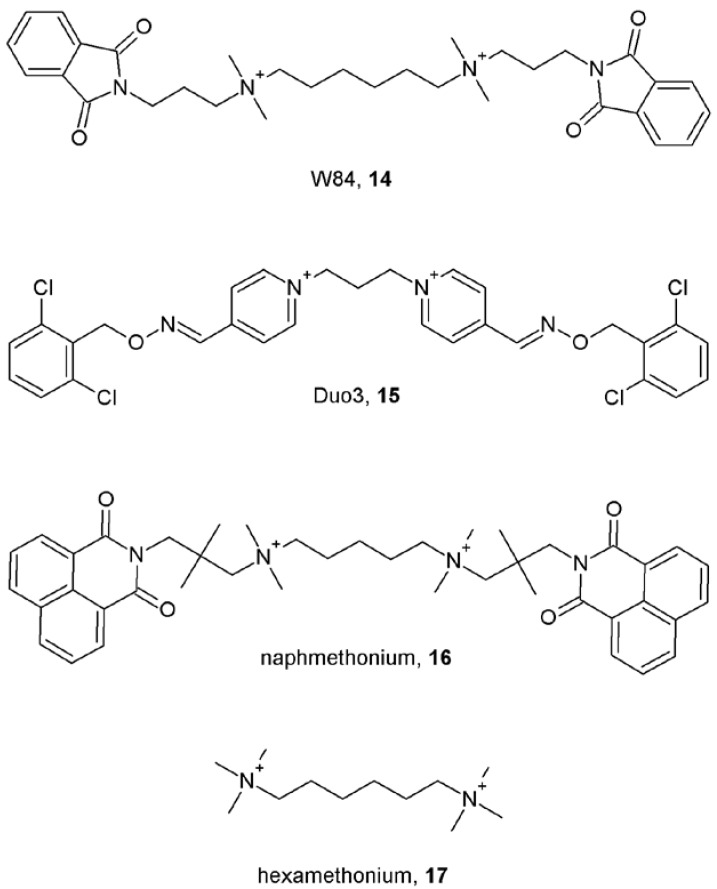
Structures of symmetric allosteric modulators of muscarinic receptors.

As can be seen allosteric modulators of muscarinic receptors vary in chemical nature and physiological properties. Both steroidal (pancuronium [31], rapacuronium [36]) and non-steroidal neuromuscular blockers [31] have been shown to allosterically modulate binding and function of muscarinic receptors. Besides various channel blockers and ligands of acetylcholine binding proteins, additional identified allosteric modulators of muscarinic receptors include peptides like dynorphin [37], relatively large (m.w. > 700 Da) molecules like toxiferous alkaloids [38] and small molecules like strychnos and vinca alkaloids [39] (Figure 2), antibiotics like staurosporine [40], endogenous metabolites like thiochrome [41] *etc*. Thus, the nature of muscarinic allosteric modulators is very diverse and finding a common simple feature is very hard, as exceptions exist for every single feature. All known muscarinic allosteric modulators contain two or more nitrogen atoms. While in the majority of modulators two of these nitrogen atoms are 400 to 500 pm apart, there are exceptions like gallamine (**1**, Figure 1) where in its energetically favorable conformation in water the distance between the nitrogen atoms is about 800 pm, or like pancuronium and rapacuronium, where the distance between the nitrogen atoms is more than 1,100 pm. While most of muscarinic allosteric modulators contain one or more atoms of oxygen, tacrine (**7**, Figure 1) does not contain any. Several modulators are symmetrical molecules [alcuronium (**2**) and obidoxime (**6**) (Figure 1), toxiferine I (**8**) and caracurine V (**9**) (Figure 2), W-84 (**14**) and Duo-3 (**15**) (Figure 3)]. Their “halves” retain allosteric properties and selectivity, albeit with lower affinity.

Detailed SAR and 3D QSAR analysis showed that spatial orientation of aromatic indole rings of the caracurine V gives better spatial orientation for binding to M_2_ receptors than tetrahydrooxepine rings of toxiferine that leads to 10-fold higher binding affinity [42]. The wide range of binding affinities of bis-quaternary analogues of caracurine V (**9**; Figure 2) could be explained by steric and electrostatic properties of *N*-substituents. Namely, non-polar dimethyl, diallyl and dipropargyl *N*-substituents in caracurine V increased binding affinity to NMS-occupied M_2_ receptor up to 100-fold [42]. Taken together, binding affinity of allosteric modulators is given by a wide-range of features that require good 3D models to comprehend. To complicate the situation even more not all allosteric modulators bind to the same binding site (see below) [40] and thus SAR differ among pharmacophores. 

## 6. Binding Site of Allosteric Modulators at Muscarinic Receptors

**Figure 4:**
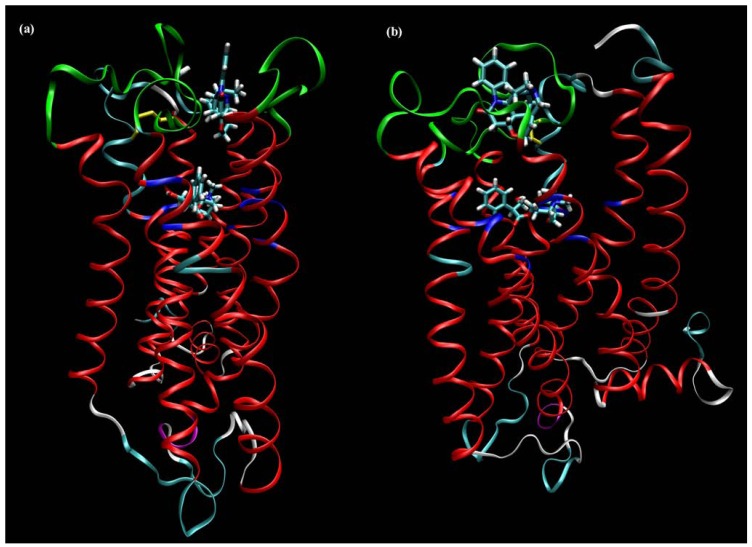
Location of orthosteric and allosteric binding sites on a homology model of M_2_ muscarinic receptor. Orientation, Extracellular, top; **(a)** TM5, front; **(b)** TM7, front. Structure, α-helices, red; 3.10 helices, purple; coil, white; turn, cyan; disulfide bridge, yellow; orthosteric site, blue; allosteric site, green. Ligands, orthosteric site, NMS; allosteric site, strychnine.

Charged cell-impermeable allosteric modulators work well on whole cells and tissues. Therefore, the extracellular parts of muscarinic receptors became prime focus in the search for binding sites of allosteric modulators. Another evidence for binding of allosteric modulators to the extracellular domains of muscarinic receptors is the observation that even supra-saturating concentrations (that do not bring further change in affinity) of alcuronium slow down both association and dissociation of orthosteric antagonist NMS in a concentration-dependent manner [43], suggesting that binding of alcuronium induces steric hindrance of the access of NMS to the orthosteric binding site. Among the five muscarinic receptor subtypes, the M_2_ subtype has the highest affinity for the prototypical allosteric modulators gallamine and alcuronium (Figure 1), whereas affinities of the M_3_ subtypes for these modulators are low [44,45]. Replacing potentially important residues in the extracellular domain unique for M_2_ receptors revealed importance of negatively charged amino acids in the EDGE motif [46] and adjacent amino acids (Val168, Tyr177, M_2_ sequence) [47] of the second extracellular (ο2) loop of M_2_ receptor for high affinity binding of gallamine. These amino acids were shown to be important also for the structurally different allosteric modulators alcuronium [47] and W-84 (Figure 3) [48]. Because gallamine binds to all five muscarinic subtypes 21 conserved residues in the extracellular domain of M_1_ receptor were mutated to alanine to identify the gallamine binding site. Mutations of tryptophan 400 at the o3 loop and tryptophan 101 at the junction of the third transmembrane domain (TM3) and the o2 loop to alanine inhibited gallamine binding, respectively [49]. The role of the o3 loop in determining the affinity and binding cooperativity of gallamine, alcuronium [47] and strychnine-like compounds [50] has been reported. Also the requirement of epitopes at the junction of o3 and TM7 and o2 and TM4 and TM5 was demonstrated for high-affinity binding of alkane-bisammonium (Figure 3) and caracurine V (**9**; Figure 2) type allosteric ligands to M_2_ receptors [51]. Furthermore, a cluster of tryptophans and threonine residues at the top of TM7 were shown to play a critical role in affinity and subtype selectivity of alkane-bisammonio compounds W84 (**14**) and naphmethonium (**16**) (Figure 3) and gallamine (**1**; Figure 1) but not diallylcaracurine V (**9**; Figure 2) [52]. Docking simulations of these compounds to a homology model of the M_2_ receptor showed that aromatic moieties of alkane-bisammonio compounds might be fixed in a sandwich like manner by π-π interactions between tryptophan at the top of TM7 and tyrosine in the middle of the o2 loop [52]. Taken together, these allosteric modulators bind to a relatively large domain between the o2 and o3 loops and their binding site also involves amino acids at junctions of these loops and TM domains (Figure 4).

**Figure 5:**
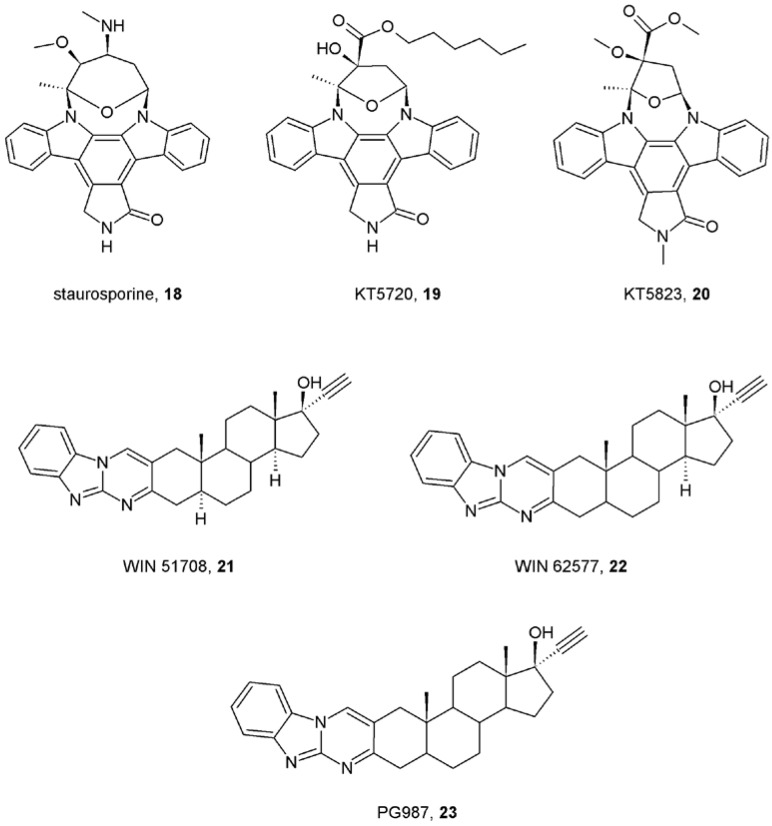
Structures of “atypical” allosteric modulators of muscarinic receptors.

In addition to this well-studied “common” allosteric binding site, a second site was revealed pharmacologically [40,54]. Staurosporine (**18**) and its analogues KT5720 (**19**) and KT5823 (**20**) (Figure 5) displayed allosteric interaction with the orthosteric ligands NMS and acetylcholine and interacted with the prototypical allosteric modulators gallamine (**1**; Figure 1) or brucine (**11**; Figure 2) in a non-competitive manner [40]. Similarly, the neurokinin receptor antagonists WIN 62,577 (**21**) and WIN 51,708 (**22**) (Figure 5) bind in micromolar concentrations to muscarinic receptors competing with saturosporine and KT5720 and interacting with gallamine and strychnine in a non-competitive manner [54]. Interestingly, while prototypical muscarinic allosteric modulators strongly decelerate [^3^H]NMS dissociation [43,44,45] the WIN compounds had little or no effect on [^3^H]NMS dissociation and their analogue PG987 (**23**; Figure 5) even accelerated it. Further studies confirmed allosteric interaction between WIN compounds and alcuronium or brucine on receptors in the absence of orthosteric ligands [55]. However, the location of this second allosteric binding site has not been identified yet. To make things even more complicated, work with the allosteric radioligand [^3^H]dimethyl-W84 showed that a “common” allosteric site (located between o2 and o3 loops) can accommodate two molecules of allosteric modulator(s) [e.g. two molecules of tacrine (**7**; Figure 1) or one molecule of tacrine and one molecule of hexamethonium (**17**) (Figure 3)] and that the “atypical” allosteric modulator Duo3 (**15**; Figure 3) may interact not only with the receptor “common” allosteric site, but also interacts in different binding modes [53]. Therefore it is more appropriate to consider the allosteric binding site as a domain with which allosteric modulators interact in various ways and with various subsets of amino acids in this domain.

**Figure 6:**
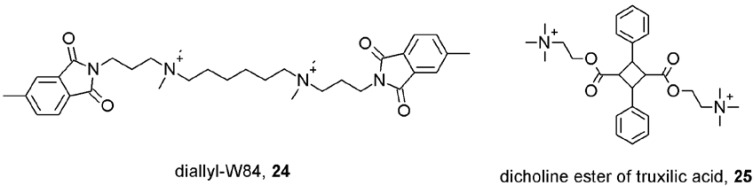
Structures of radiolabeled allosteric modulators of muscarinic receptors.

Determination of the location of the allosteric binding site was mostly done by indirect measurements via allosteric interaction between allosteric modulators and radiolabeled orthosteric antagonists. Location of and especially analysis of interactions at the allosteric binding site could be facilitated by a radiolabeled allosteric modulator that would allow for more direct screening of the putative binding site via simple competition binding assays. However, the only existing radiolabeled muscarinic allosteric modulator with sufficient affinity found is [^3^H]dimethyl-W84 (**24**; Figure 6) [56]. Other candidates for radiolabeling, α-truxillic acid esters (**25**; Figure 6) [57], exert unacceptable non-specific binding.

## 7. Mechanisms of Conformational Changes Induced by Allosteric Modulators

Although the relationship between structure and binding affinity is known at least for some groups of allosteric modulators [42,58,59] virtually nothing is known about the mechanisms of the conformational changes that allosteric modulators induce. This could be explained by two main reasons. First, there are only few papers primarily analyzing conformational changes induced by allosteric modulators. Second, allosteric interactions are very complex and depend on specific combinations of receptor, orthosteric ligand and allosteric modulator, where mere change of one of these may lead to a completely different sort of cooperativity. For example, while acetylcholine has negative binding cooperativity with strychnine at M_3_ receptors, it has slightly positive binding cooperativity with strychnine's dimethoxy analogue brucine (**11**; Figure 2) [39] that can be strongly enhanced by simple chloration at the methyl group [60]. In contrast, while *N*-substituents at the caracurine V (**9**; Figure 2) pharmacophore dramatically changed affinity, the binding cooperativity of these allosteric modulators remained mainly unchanged [42].

On the contribution of structure of orthosteric ligands to cooperativity it has been shown that cooperativity with alcuronium on M_2_ receptors is dependent on the distance between the nitrogen and carboxyl C atoms. Othosteric antagonists with N to carboxyl C distances over 500 pm (NMS, 4-diphenylacetoxy-*N*-dimethylpiperidium, and *N*-methylpiperidyl benzylate) displayed positive, while orthosteric ligands with N to carboxyl C distances less than 500 pm (*N*-methylquinuclidinyl benzilate, quinuclidinyl benzylate, and acetylcholine) displayed negative binding cooperativity with alcuronium (**3**; Figure 1) [61]. According to docking of orthosteric ligands to homology models of the M_2_ receptor and knowledge from mutagenesis studies, the nitrogen group of orthosteric ligands interacts with aspartic acid in the TM3 domain and the carboxyl group of orthosteric ligands interacts with residues in the TM6 domain [62]. Thus data suggest that allosteric modulators change orientation of the TM3 and TM6 domains. Probably alcuronium moves them apart so that the orthosteric binding site becomes larger and therefore more favorable for ligands with longer distance between the N and carboxyl C atoms but less favorable for ligands with shorter distance between the N and carboxyl C atoms. The idea of helical bundle rearrangement is further supported by findings that disruption of a conserved disulfide bond between the top of the TM3 domain and middle of the o2 loop attenuates cooperativity between allosteric modulators and orthosteric ligands [63,64]. Disruption of this disulfide bond makes the o2 loop more flexible and thus weakens transfer of the conformational changes to the helical bundle.

The contribution of receptor structure to the nature of binding cooperativity is also complicated. Unlike for affinity, not a single key amino acid can be pin-pointed to determine the type and strength of cooperativity. Rather, multiple receptor domains and amino acids scattered within a receptor domain are responsible for the type and strength of cooperativity. Mutation of amino acids in the o3 loop of the M_3_ receptor (on which NMS and alcuronium exert negative cooperativity) to corresponding residues of the M_2_ sequence (on which NMS and alcuronium exert positive cooperativity) led to gradual change from negative to positive cooperativity between NMS and alcuronium [47]. If the type and degree of binding cooperativity is given by TM3 and TM6 rearrangement and if alcuronium binds between the o2 and o3 loops then these data suggest that mutations in the o3 loop (connecting the TM6 and TM7 domains) lead to rearrangement of the TM6 domain in respect to the rest of the receptor. This is in accordance with the notion that M_2_ receptors have lower affinity for NMS than M_3_ receptors and mutations in the o3 loop gradually decreased affinity for NMS [47]. Interestingly, at the same chimera (M_3_ receptor with the o3 loop mutated to M_2_ sequence) NMS had positive cooperativity with Wieland-Gumlich's aldehyde (WGA) although both wild types display negative cooperativity [50]. These data indicate that the binding cooperativity at muscarinic receptors is not dictated only by rearrangement of TM3 and TM6 but also other factors come into play. There may be interplay between the o2 and o3 loops as supported by a shift in binding cooperativities between strychnine-like modulators (Figure 2) and NMS to more positive values also by mutations in the o2 loop [50]. Alternatively, mutations in the o3 loop may lead to changes in the WGA binding mode that leads to conformational changes not observed in any of the wild types.

## 8. Endogenous Allosteric Modulators of Muscarinic Receptors

**Figure 7:**
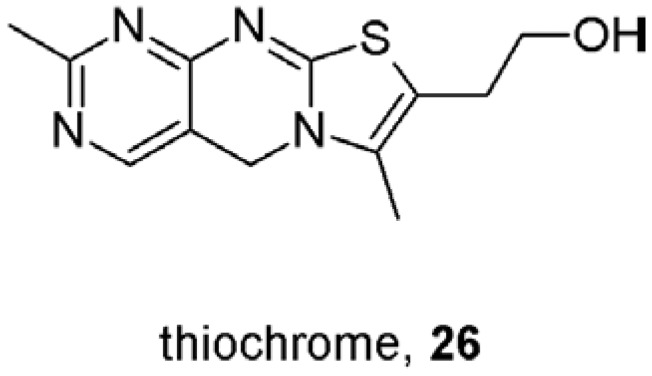
Structure of thiochrome.

As can be seen from the above discussion the domain on the muscarinic receptors that can bind allosteric modulators is relatively large and the chemical nature of allosteric modulators is multifarious. Thus the odds for the existence of endogenous allosteric modulator(s) are high. Actually, it has been shown in several laboratories that the supernatant fraction prepared from animal tissues contains an endogenous inhibitory factor (EIF) that allosterically decreases binding of both antagonists and agonists. An acidic, moderately heat-stable, low molecular weight, probably of peptide nature EIF was prepared from calf hearts [65], rat thymocytes [66], rat heart [67], human brain [68] and guinea-pig illeum [69]. Although EIF was partially purified and its properties characterized [69] it has never been identified. Interestingly, studies on muscarinic receptors that do not deal with purification and identification of endogenous allosteric modulator of muscarinic receptors offer several alternative candidates for endogenous allosteric modulators of muscarinic receptors. These are eosinophil major basic protein [70], dynorphin and myelin basic protein [37], arachidonic acid [71] or thiochrome (Figure 7), a metabolite of thiamine [41]. However, these compounds do not have physiochemical properties of the abovementioned EIF. Importantly, this EIF has been shown to take a part in the development of Alzheimer’s disease [72] where in complex with other proteins and small non-protein molecules represents a risk factor catalysing oxidative stress in Alzheimer’s brains [73]. Thus, an additional potential advantage of positive allosteric modulators of acetylcholine action would be competing with EIF at muscarinic receptors and canceling its undesirable effects.

## 9. First Positive Allosteric Modulators of Acetylcholine

The Holy Grail in treatment of impaired neurotransmission is to preserve its time and space pattern. Selective allosteric potentiators of acetylcholine at muscarinic receptors would serve this role in cognitive deficits and other aspects connected with cholinergic transmission in Alzheimer’s and schizophrenia. In theory, potentiation of neurotransmitter action can be achieved by increasing affinity of the receptor for the neurotransmitter or by increasing efficiency of coupling of the receptor to G-protein upon activation by the neurotransmitter. Allosteric modulators with positive cooperativity with acetylcholine binding and/or function at one subtype (M_1_ in Alzheiner’s and M_4_ in schizophrenia) and neutral cooperativity at other subtypes would potentiate acetylcholine action only at these subtypes. Most importantly, this takes place only when and where acetylcholine is released, in a fashion that preserves the time and space pattern of acetylcholine action. Early proof-of-concept studies by several laboratories were successful in identifying positive allosteric modulators of acetylcholine binding at M_1_, M_3_ and M_4_ receptors. Brucine (**11**; Figure 2) displayed weak positive cooperativity in binding with acetylcholine at the M_1_ and M_3_ receptors [39]. Interestingly, brucine analogues *N*-chloromethyl brucine and brucine-*N*-oxide displayed positive cooperativity with acetylcholine binding at M_3_ receptors but neutral to negative cooperativity at M_1_ receptors [60]. Structurally different from brucine the sterol based compound WIN 62,577 (**22**; Figure 5) increased affinity of acetylcholine at M_3_ receptors almost 2-fold [54]. Its analogues displayed different types of cooperativity and both higher and lower potency rendering determination of structure-activity relationship unfeasible. Thiochrome (**26**; Figure 7) is an oxidation product and metabolite of thiamine that binds to all five muscarinic receptor subtypes with micromolar affinity. Interestingly, it displayed positive cooperativity with acetylcholine exclusively at M_4_ receptor [41]. Its cooperativity with acetylcholine at other muscarinic subtypes was neutral thus its effects on acetylcholine binding and action can be considered absolutely selective. However, these compounds lacked efficacy and physiochemical properties in *in vivo* studies.

## 10. Truly Selective Positive Allosteric Modulators of Acetylcholine

**Figure 8:**
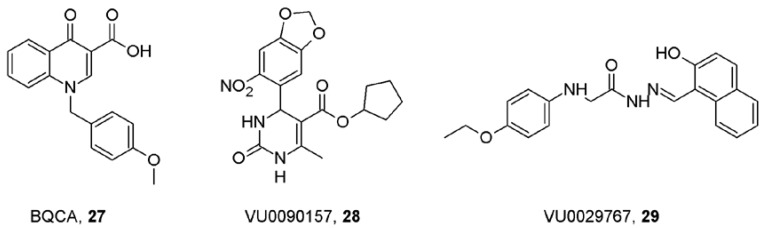
Structure of M_1_ selective allosteric modulators.

A major breakthrough in the research for muscarinic allosteric modulators was the discovery of benzylquinoline carboxylic acid (BQCA, **27**; Figure 8), the positive allosteric modulator of acetylcholine binding and action at M_1_ receptors that positively regulates non-amyloidogenic APP processing *in vitro* [74,75]. Besides the expected procognitive effects BQCA also increased blood flow to cerebral cortex that is beneficial in neurodegenerative diseases like Alzheimer's disease. These findings support the hypothesis that it will be possible to develop highly selective allosteric potentiators with procognitive effects. Recently, multiple novel M_1_ selective positive allosteric modulators have been identified in a high-throughput functional-screening [76]. These compounds belong to several structurally diverse pharmacophores. None of them had agonistic activity and behaved as pure allosteric modulator of acetylcholine function in the following manner: (1) increased acetylcholine potency without change in efficacy; (2) did not compete with acetylcholine binding. The most selective compounds were VU0090157 (**28**) and VU0029767 (**29**) (Figure 8). 

At M_1_ receptors both compounds increased binding affinity for acetylcholine and potentiated activation of PLC. Interestingly, VU0090157 also potentiated activation of PLD but VU0029767 did not, demonstrating signal trafficking and activation bias by structurally heterologous compounds. This screening also yielded the first highly M_5_ selective ligand VU0238429 that is an allosteric potentiator of acetylcholine action at this receptor [77].

**Figure 9:**
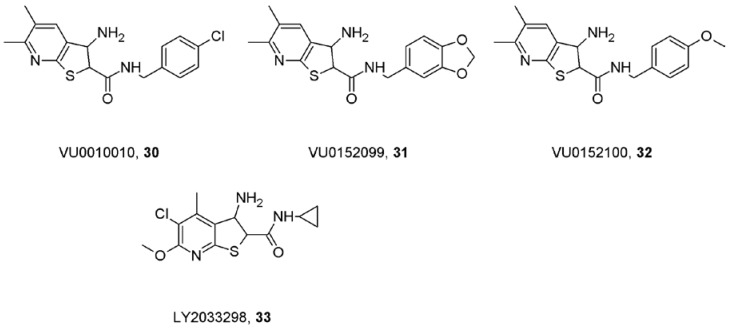
Structure of M_4_-selective allosteric modulators.

An important point in search for an M_4_ selective positive allosteric modulator of acetylcholine function was the identification of the pharmacophore VU0010010 (**30**; Figure 9) [78]. This compound increased affinity for actetylcholine at M_4_ receptors and increased efficiency of coupling of M_4_ with G-proteins *in vitro* but did not activate the receptors in the absence of agonist. *In vivo,* VU0010010 facilitated M_4_-mediated autoinhibition of acetylcholine release in the hippocampus but had no effect on responses mediated by M_1_ or M_2_ receptors. Compounds VU0152099 (**31**) and VU0152100 (**32**; Figure 9) that are based on the VU0010010 phamacophore retain M_4_ selectivity and on top of it readily cross the blood-brain barrier and have improved pharmacokinetic properties over the parent compound [79]. At the same time, the M_4_-selectivivity of a structurally different allosteric potentiator of acetylcholine function, LY2033298 (**33**; Figure 9), was reported [80]. This compound potentiated acetylcholine-induced GTPγS binding and FLIPR signal *in vitro* only at M_4_ receptors. In rats it reduced the conditioned avoidance response, another paradigm predictive of antipsychotic drug efficacy. In mutant M_4_ receptors insensitive to activation by acetylcholine but increased sensitivity to activation by clozapine-like compounds, LY2033298 caused functional rescue of acetylcholine potency and efficacy demonstrating the multitude of ways to activate the receptor [81]. Importantly, unlike other allosteric modulators (see part 7), amino acids governing binding cooperativity between LY2033298 and acetylcholine were recently identified at the junction of TM2 and o1 loop [82].

## 11. Caveats about Allosteric Modulators

The major caveat of allosteric modulation of neurotransmitter action is that it is not always in concert with neurotransmitter binding. Our current study shows that although the allosteric modulator rapacuronium strongly decreases the overall affinity of M_3_ receptors muscarinic acetylcholine receptors for acetylcholine it accelerates its rate of binding. The latter leads to facilitation of its action *in vitro* [83]. This explains the severe rapacuronium-induced brochospasm observed *in vivo* [84]. The time between acetylcholine release and termination of its action by acetylcholinesterase is in the range of a fraction of a second. Therefore, the effects of allosteric modulators in the early non-equilibrium stage of receptor signaling are therapeutically more important than effects on acetylcholine equilibrium binding, as the latter conditions do not occur *in vivo*. Therefore it is necessary to employ fast functional assays in screening for potential allosteric modulators of neurotransmission that much better simulate physiological conditions than long-lasting equilibrium binding experiments.

Also, the prototypic allosteric modulators of muscarinic receptors alcuronium, gallamine, and strychnine weakly stimulated production of inositol phosphates in CHO cells expressing the M_1_ or the M_3_ receptors and inhibited synthesis of cAMP in CHO cells expressing the M_2_ or the M_4_ receptors in the absence of receptor agonists [85] although they display negative cooperativity with acetylcholine. This study implies that: (1) an allosteric modulator by itself may possess concomitant agonistic properties; (2) conformations with low affinity for the neurotransmitter may represent an additional active state of the receptor so that there are multiple active conformations and multiple ways to activate the receptor. Thus, our understanding of the mode of receptor activation becomes more complex and makes the search for allosteric modulators more difficult. Specifically, it is essential to control for concomitant agonistic activity of potential allosteric modulators in functional screening.

**Figure 10:**
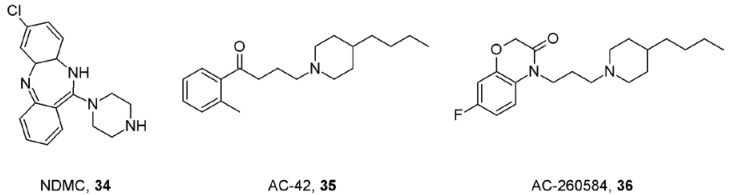
Structures of muscarinic ectopic agonists.

The possibility to activate muscarinic receptors from the allosteric binding domain in combination with the subtype diversity of this domain allowed for discovery of highly selective muscarinic agonists, for example the M_1_/M_4_-preferring agonist *N*-desmethylclozapine (NDMC, **34**; Figure 10) [86], the M_1_-selective full agonist AC-42 (**35**; Figure 10) [87] and its analogue AC-260584 (**36;** Figure 10) that display increased pro-cognitive action and oral bioavailability [88]. The allosteric mode of action of these compounds was proven by mutations that render the M_1_ receptor insensitive to activation by acetylcholine but do not alter the activity of AC-42 or NDMC. On the other hand, the activity of AC-42 and NDMC can be eliminated by mutations in the TM1 and TM7 domains that do not alter receptor activation by acetylcholine [89,90]. Strictly speaking, however, these compounds should be termed ectopic agonists as they bind “on top” of the orthosteric binding site but not to the classic allosteric binding site. Interestingly, unlike orthosteric agonists, prolonged exposure of CHO cells to AC-42 did not alter either cell-surface or total cellular M_1_ receptor expression [91]. Theoretically, preventing receptor desensitization and down-regulation could be another advantage of putative positive allosteric modulators of acetylcholine action.

## 12. Conclusions

Since the discovery of gallamine as the first allosteric modulator of muscarinic receptors 30 years ago our understanding of binding, action and structure-activity relationship of muscarinic allosteric modulators has undergone huge progress. Despite of this increased knowledge, high-throughput functional screening still remains a better approach in the search for positive allosteric modulators of acetylcholine action than knowledge-based design. Recent discoveries of selective positive allosteric modulators of acetylcholine with therapeutic potential in the treatment of psychiatric and neurologic disorders like Alzheimer’s or schizophrenia are encouraging. These compounds represent a new and better way to treat these debilitating disorders and a new hope for suffering ones and their families.

## Notes (List of compounds and their chemical names)

**Gallamine (1)**: 2-[2,6-bis(2-diethylaminoethyloxy)phenoxy]-*N,N*-diethylethanamine.

**Pancuronium **(**2**): [(2*S*,3*S*,5*S*,8*R*,9*S*,10*S*,13*S*,14*S*,16*S*,17*R*)-3-acetyloxy-10,13-dimethyl-2,16-bis(1-methylpiperidin-1-ium-1-yl)-2,3,4,5,6,7,8,9,11,12,14,15,16,17-tetradecahydro-1*H*-cyclopenta[a]phen-anthren-17-yl] acetate.

**Alcuronium **(**3**): 2-[14,30-diallyl-36-[2-hydroxy-(*E*)-ethylidene]-14,30-diazonia-8,24-diazaundeca-cyclo-[25.5.2.2^11,14^.1^8,26^.1^10,24^.0^1,37^.0^2,7^.0^13,17^.0^17,38^.0^18,23^.0^30,33^]octatriaconta-2(7),3,5,9,18(23),19,21,25-octaen-28-yliden]-1-ethanol.

**Verapamil (4)**: 2-(3,4-dimethoxyphenyl)-5-[2-(3, 4-dimethoxyphenyl)ethylmethylamino]-2-propan-2-ylpentanenitrile.

**4-aminopyridine (5);** pyridin-4-amine.

**Obidoxime (6)**: oxo-[[1-[[4-(oxoazaniumylmethylidene)pyridin-1-yl]methoxymethyl]pyridin-4-ylide-ne]methyl]azanium.

**Tacrine (7)**: 1,2,3,4-tetrahydroacridin-9-amine.

**Toxiferine I (8)**: 2-[37-[2-hydroxy-(E)-ethylidene]-14,30-dimethyl-8,14,24,30-tetraazaundecacyclo-[25.5.2.2^11,14^.1^1,8^.1^10,17^.0^2,7^.0^13,17^.0^24,25^.0^26,38^.0^18,23^.0^30,33^]octatriaconta-2(7),3,5,9,18,20,22,25-octaen-28-yliden]-1-ethanol.

**Caracurine V (9)**: 2-[(1R,9R,16S,18R,19R,20S,21R,29Z,31S,32E,37S,41S)- 10-oxa-8,15,28,34-tetra-azadodecacyclo[29.5.2.2^15,21^.1^1,8^.0^2,7^.0^9,19^.0^13,18^.0^16,21^.0^20,28^.0^22,27^.0^34,37^.0^30,41^]hentetraconta-2,4,6,12,22, 24,26,29-octaen-32-ylidene]ethan-1-ol; ethanol, 2-[(3aR,9Z,9bS,10S,11aS,14aR,19aR,23aR,23bR, 23cS,24aS)-2,3,11,11a,13,14,21,23a,23b,23c,24,24a-dodecahydro-10,12-ethano-1,23-methano-9bH, 10H,19aH-indolo[1',2',3':1,10]oxepino[2',3',4':6,7]pyrr.

**Strychnine (10)**: (1R,11S,18S,20R,21R,22S)-12-oxa-8,17-diazaheptacyclo-[15.5.2.0^1,18^.0^2,7^.0^8,22^.0^11,21^. 0^15,20^]tetracosa- 2,4,6,14-tetraen-9-one.

**Brucine (11)**: (1R,11S,18S,20R,21R,22S)-4,5-dimethoxy-12-oxa-8,17- diazaheptacyclo-[15.5.2.0^1,18^. 0^2,7^.0^8,22^.0^11,21^.0^15,20^]tetracosa- 2,4,6,14-tetraen-9-one

**Vincamine (12)**: methyl (15S,17S,19S)-15-ethyl-17-hydroxy-1,11- diazapentacyclo-[9.6.2.0^2,7^.0^8,18^. 0^15,19^]nonadeca- 2,4,6,8(18)-tetraene-17-carboxylate

**Eburnamonine (13)**: (15S,19S)-15-ethyl-1,11- diazapentacyclo[9.6.2.0^2,7^.0^8,18^.0^15,19^]nonadeca- 2,4,6,8(18)-tetraen-17-one.

**W-84 (14)**: 3-(1,3-dioxoisoindol-2-yl)propyl-[6-[3-(1,3-dioxoisoindol-2-yl)propyl-dimethylazanium-yl]hexyl]-dimethylazanium.

**Duo3 (15)**: 1,1'-propane-1,3-diylbis{4-[(E)-{[(2,6-dichlorobenzyl)oxy]imino}methyl]-1-ethylpyridi-um.

**Naphmethonium (16)**: 3-[3-({5-[(3-{2,4-dioxo-3- azatricyclo[7.3.1.05,13]trideca- 1(12),5,7,9(13),10-pentaen-3-yl}-2,2- dimethylpropyl)dimethylamino]pentyl}dimethylamino)- 2,2-dimethylpropyl]-3- azatricyclo[7.3.1.05,13]trideca- 1(12),5,7,9(13),10-pentaene-2,4-dione.

**Hexamethonium (17)**: trimethyl-[6-(trimethylazaniumyl)hexyl]azanium.

**Staurosporine (18)**: 3-methoxy-2-methyl-4-(methylamino)-29-oxa-1,7,17-triazaoctacyclo[12.12.1^2,6^. 0^7,28^.0^8,13^.0^15,19^.0^20,27^.0^21,26^]nonacosa-8,10,12,14,19,21,23,25,27-nonaen-16-one.

**KT5720 (19)**: 9,12-Epoxy-1H-diindolo[1,2,3-fg:3',2',1'-kl]pyrrolo[3,4-i][1,6]benzodiazocine-10-carb-oxylic acid, 2,3,9,10,11,12-hexahydro-10-hydroxy-9-methyl-1-oxo-, hexyl ester, (9R,10R,12S)-rel.

**KT5823 (20)**: 9-methoxy-9-methoxycarbonyl-8-methyl-2,3,9,10-tetrahydro-8,11-epoxy-1H,8H,11H-2,7b-11a-triazadibenzo(a,g)cycloocta(cde)-trinden-1-one.

**WIN 51708 (21)**: 17-β-hydroxy-17-α-ethynyl-5-α-androstano[3,2-b]pyrimido[1,2-a]benzimidazole.

**WIN 62577 (22)**: 17-β-hydroxy- 17-α-ethynyl-δ(4)-androstano[3,2-b]pyrimido[1,2-a]benzimidazole.

**PG987 (23)**: 7-β-hydroxy-17-α-δ(4)-androstano[3,2-b]pyrido[2,3-b]indole.

**Dimethyl-W84 (24)**: 2-[2,4-diphenyl-3-[2-(trimethylazaniumyl)ethoxycarbonyl]cyclobutanecarbonyl]

oxyethyl-trimethylazanium.

**Truxilic acid dicholine ester (25)**: 2-[2,4-diphenyl-3-[2-(trimethylazaniumyl)ethoxycarbonyl]cyclo-butanecarbonyl]oxyethyl-trimethylazanium.

**Thiochrome (26)**: 2-(2,7-dimethyl-5H-pyrimido[4,5-d][1,3]thiazolo[3,2-a]pyrimidin-8-yl)ethanol.

**Benzylquinoline carboxilic acid (27)**: 1-(4-methoxy)-4-oxo-1,4-dihydroquioline-3-carboxylic acid.

**VU0090157 (28)**: cyclopentyl-1,6dimethyl-4-(6nitrobenyo[d][1,3]dioxol-5-yl)-2-oxo-1,2,3,4-tetrahyd-ropyrimidine-5-carboxylate.

**VU0029767 (29)**: (E)-2-(4-ethoxyphenylamino)-N'-((2-hydroxynaphthalen-1-yl)methylene) aceto-hydrazide.

**VU0010010 (30)**: 3-amino-N-(4-chlorobenzyl)-4,6-dimethylthieno[2,3-b]pyridine-2-carboxamide.

**VU0152099 (31)**: 3-amino-N-(1,3-benzodioxol-5-ylmethyl)-5,6-dimethyl-2,3-dihydrothieno[2,3-b] pyridine-2-carboxamide.

**VU0152100 (32)**: 3-amino-N-(4-methoxybenzyl)-5,6-dimethyl-2,3-dihydrothieno[2,3-b]pyridine-2-carboxamide.

**LY2033298 (33)**: 3-amino-5-chloro-6-methoxy-4-methyl-thieno[2,3-b]pyridine-2-carboxylic acid cyclopropylamide.

**N-desmethylclozapine (34)**: 3-chloro-6-piperazin-1-yl-5H-benzo[c][1,5]benzodiazepine.

**AC-42 (35)**: 4-n-Butyl-1-[4-(2-methylphenyl)-4-oxo-1-butyl]-piperidine.

**AC-260584 (36)**: (4-[3-(4-butylpiperidin-1-yl)-propyl]-7-fluoro-4H-benzo[1,4]oxazin-3-one).
